# Establishment of a minor groove binder-probe based quantitative real time PCR to detect *Borrelia burgdorferi *sensu lato and differentiation of *Borrelia spielmanii *by *osp*A-specific conventional PCR

**DOI:** 10.1186/1756-3305-3-69

**Published:** 2010-08-10

**Authors:** Christina Strube, Victor M Montenegro, Christian Epe, Elke Eckelt, Thomas Schnieder

**Affiliations:** 1 Institute for Parasitology, University of Veterinary Medicine Hannover, Buenteweg 17, 30559 Hannover, Germany; 2 Laboratorio de Parasitología, Escuela de Medicina Veterinaria, Universidad Nacional, Costa Rica, PO Box 304-3000, Heredia, Costa Rica; 3 Current Address: Novartis Centre de Recherche Santé Animale SA, CH-1566 St. Aubin FR, Switzerland

## Abstract

**Background:**

*Borrelia burgdorferi *sensu lato (sl), the causative agent of Lyme borreliosis, is transmitted by ticks of the genus *Ixodes *as vector. For identification of *Borrelia *infections in ticks a TaqMan™ minor groove binder (MGB) probe-based quantitative real time PCR (qPCR) was established targeting the 5S-23S intergenic spacer. Extension to a duplex qPCR included an *Ixodes *spp. positive control to verify successful DNA isolation. Besides qPCR, an *osp*A-specific conventional PCR for species-specific identification of *B. spielmanii *was established. Afterwards 1000 *I. ricinus *flagged in the city of Hanover, Germany, were investigated for *B. burgdorferi *sl infections followed by species identification. Furthermore, *I. hexagonus *ticks were investigated to proof applicability of the PCRs.

**Results:**

Quantitative real time PCR (qPCR) identifying *B. burgdorferi *sl in ticks was able to detect 1-10 copies per reaction. *B. spielmanii osp*A-specific conventional PCR was also highly specific and showed no cross reactions with the other tested *Borrelia *species. From 1000 hanoveranian ticks 24.3% were positive compared to only 7.4% positives by dark-field microscopy. Related to tick stage 1.7% larvae, 18.1% nymphs, and 34.6% adults were positive. The most frequent species was *B. garinii*, followed by *B. afzelii*, *B. spielmanii*, *B. valaisiana *and *B. burgdorferi *sensu stricto (ss). 70.6% of *I. ricinus *were mono-infected, whereas 28.0% and 1.4% were infected with two and three *Borrelia *species, respectively. From 232 *I. hexagonus *collected from hedgehogs in different sites of Germany, qPCR detected 5.7% to be infected with *B. burgdorferi *sl, which were identified as *B. afzelii*, *B. garinii *and *B. spielmanii*.

**Conclusions:**

The evaluated qPCR to detect *B. burgdorferi *sl in *Ixodes *spp. is highly specific and sensitive. As a duplex qPCR including detection of *Ixodes *spp. DNA it is the first DNA based technique incorporating a control for successful DNA isolation from the vector tick. Establishment of a *B. spielmanii *specific conventional PCR filled the gap in PCR identification of principal European *Borrelia *genospecies. Practical application showed that all European pathogenic *Borrelia *spp. were present in *I. ricinus *flagged in recreational areas of the city of Hanover and confirmed *I. hexagonus *as reservoir for pathogenic *Borrelia *spp.

## Background

Ticks of the genus *Ixodes *are transmission vectors for *Borrelia burgdorferi *sensu lato (sl), the causative agent of Lyme borreliosis (LB). In Central Europe, the sheep or forest tick *I. ricinus *is the main vector for *B. burgdorferi *sl with a distribution area between the 66th and 39th degree of latitude and from Portugal to Russia [1]. Besides this classical vector, the hedgehog tick *I. hexoganus*, the fox tick *I. canisuga*, and the sea bird tick *I. uriae *contribute to the circulation of *B. burgdorferi *sl in Europe [2-4]. The *B. burgdorferi *sl-complex comprises worldwide today at least 15 spirochete species, namely *B. burgdorferi *sensu stricto (ss), *Borrelia afzelii*, *Borrelia andersonii*, *Borrelia bissettii*, *Borrelia californiensis*, *Borrelia garinii*, *Borrelia japonica*, *Borrelia lusitaniae*, *Borrelia sinica*, *Borrelia spielmanii*, *Borrelia tanukii*, *Borrelia turdi*, *Borrelia valaisiana *as well as the recently described *Borrelia carolinensis *[5] and *Borrelia americana *[6]. Furthermore, *B. bavariensis *sp. nov. [7] formerly included in the *B. garinii *species (OspA serotype 4) and *B. yangtze *sp. nov. [8] were proposed as new species. From these, *B. burgdorferi *ss, *B. afzelii*, *B. bissettii*, *B. garinii*, *B. lusitaniae*, *B. spielmanii*, *B. valaisiana *and *B. bavariensis *sp. nov. are recognized in Central Europe. While *B. burgdorferi *ss, *B. afzelii*, *B. garinii*, and *B. spielmanii *are confirmed as causative organisms of Lyme disease, the pathogenic potential of the remaining species is still unclear. However, the presence of *B. bissettii*, *B. lusitaniae*, and *B. valaisiana *in human patients [9-13] provides evidence that these species might also cause LB. While *B. burgdorferi *ss is the most common species in the United States, in Germany the pathogens *B. garinii *and *B. afzelii *are more frequent than *B. burgdorferi *ss [14-17].

In Europe, the overall mean prevalence of *Borrelia *spirochetes in ticks is 13.7% with the highest *I. ricinus *infection rates in Central Europe and a significant increase in the infection rate of adult ticks from Western to Eastern Europe [18]. In Germany, approximately 1% of larvae, 4 to 18% of nymphs and 10 to 35% of adult ticks are infected with *B. burgdorferi *sl (reviewed in [19]). Thereby prevalence variations not only depend on the region, but also on the examination methods known to exhibit unequal sensitivity and specificity values. In both cases, PCR techniques are certainly superior to other techniques used to detect *B. burgdorferi *sl.

Here we present the establishment of a highly sensitive and specific quantitative real time PCR (qPCR) based on TaqMan™ minor groove binder (MGB)-probes to detect *Borrelia burgdorferi *sl in ticks. The inference on individual *Borrelia *burden was assured by targeting a single copy region, the 5S-23S intergenic spacer [20]. A further aim was to establish a species-specific conventional PCR to discriminate *B. spielmanii *from other European genospecies. When applying the established PCR methods in a study on ticks flagged in the city of Hanover we found that all assured human pathogenic *Borrelia *species distributed in Europe were present in the collected hanoveranian ticks.

## Results

### *Borrelia burgdorferi *sl genus-specific quantitative real time PCR

Genomic DNA amplification of the 5S-23S intergenic spacer region with the genus-specific primer/TaqMan™-MGB-probe combination resulted for each tested *Borrelia *species (*B. burgdorferi *ss, *B. afzelii*, *B. garinii*, *B. valaisiana *and *B. spielmanii) *in bands of 67 bp which were verified by sequencing.

When testing specificity, no amplification products were observed by using DNA of related spirochetes or laboratory bred *I. ricinus *larvae. Regarding sensitivity, qPCR detected between 1 to10 IGS copies in the individual runs when using serial plasmid standard dilutions as template. This detection limit was also reached for the *B. spielmanii*- and *B. lusitaniae*-IGS plasmid standard showing one mismatch with the reverse primer. Since the DNA elution volume from homogenised ticks is 100 μl and 2 μl of them are used as template in qPCR, this correlates with a burden of ≥50-500 *Borrelia *individuals per tick. In this context spiking experiments revealed no negative impact of tick material on qPCR detection.

### Duplex quantitative real time PCR targeting ITS2 of *Ixodes *spp. as positive control

By testing the designed primer/TaqMan™-MGB-probe combination targeting the ITS2 sequence of *Ixodes *spp. all isolated tick DNAs (280 *I. ricinus*, 8 *I. hexagonus*, and 8 *I. canisuga*) resulted in positive qPCR signals. Based on serially diluted plasmid standards, the qPCR was able to detect 1 ITS2-copy. By using the ABsolute™ Blue QPCR low Rox Mix in the duplex qPCR reaction setup there was no obvious effect on *B. burgdorferi *sl detection whereas this detection failed when the Brilliant^® ^QPCR Master Mix was used.

Partial ITS2 sequencing of eight *I. ricinus *individuals showed 100% sequence identity with the primer-probe combination. For one *I. hexagonus *(accession no. GQ330526), a substitution (C→T) was identified within the probe sequence. Of *I. canisuga*, all individuals possessed a substitution (C→T) within the probe sequence, and one individual (accession no. GQ330529) had a second substitution (T→C). In the reverse primer sequence, an insertion (A) was observed in three individuals (accession nos. GQ330528, GQ330529, and GQ330534). Additionally, one of them showed a substitution (C→T, accession no. GQ330529). The sequence of the forward primer could not be considered since this primer was used to generate the partial *I. canisuga *ITS2 sequences.

### Evaluation of the species-specific conventional PCR

Specificity examination of the newly designed *B. spielmanii *specific *osp*A primers as well as the published *rpo*B primers for *B. burgdorferi *ss, *B. afzelii*, *B. garinii*, and *B. valaisiana *[21] using DNA of the different *Borrelia *spp. as well as from related spirochetes (*Treponema *sp. and *Leptospira *spp.) resulted in bands with the corresponding *Borrelia *spp. DNA only, no cross reactions were observed. *B. spielmanii osp*A amplification resulted in a PCR product measuring 151 bp in length and was confirmed by sequencing. Sequence alignment revealed an *osp*A forward primer identity of 82% and 69% with *B. bissettii *and *B. lusitaniae*, respectively. From the last 7 bases on the primer's 3' end at most 3 bases were identical, but not the very last one. Concerning the reverse *osp*A primer sequence identities were 85% and 76% with the above mentioned *Borrelia *spp. From the last 7 bases on the primer's 3' end at most 4 bases were identical, but not the last two ones. Further *B. spielmanii osp*A-specific primer evaluation proved negative PCR results when using DNA of 100 laboratory bred *Ixodes *larvae as template. No inhibitory effect was noticed by amplification of *B. spielmanii *DNA in presence of DNA of the other *Borrelia *species.

### Analysis of *Borrelia *infections and species distribution in ticks of the city of Hanover

Morphological determination revealed that the 1000 ticks collected in recreation areas of the city of Hanover were all belonging to the species *I. ricinus*. Stage distribution was as follows: 60 larvae, 507 nymphs, and 433 adults (229 males and 204 females). Of these, 74 were spirochete positive in dark-field microscopy (DFM), but only 54 of them were confirmed by genus-specific quantitative real time PCR (qPCR). In all, 243 ticks gave a positive qPCR signal whereas in 8 ticks the results were questionable. Table 1 shows the DFM and qPCR results in more detail. With the qPCR positive and questionable ticks species-specific conventional PCR runs were performed. In 212 out of the 243 qPCR positive ticks and in 6 out of the 8 questionable ticks the *B. burgdorferi *sl species differentiation was successful. From the 218 *Borrelia *species differentiated ticks, 154 ticks (70.6%) were mono-infected, 61 (28.0%) were infected with two and 3 (1.4%) were infected with three species of the *Borrelia burgdorferi *sl-complex. Results considering the stage distribution of the *I. ricinus *ticks are listed in Table 2. The most frequent mono-infection was with *B. afzelii *closely followed by *B. garinii *whereas *B. burgdorferi *ss caused the least common mono-infection. Frequent double-infections were the combinations *B. garinii*/*B. spielmanii*, *B. garinii*/*B. valaisiana, B. afzelii*/*B. garinii *and *B. afzelii*/*B. spielmanii*. The combinations *B. afzelii*/*B. burgdorferi *ss/*B. spielmanii *and *B. garinii*/*B. spielmanii*/*B. valaisiana *were found in triple infections. Detailed species distribution results and combinations are shown in Table 3.

**Table 1:** Distribution of *B. burgdorferi *sl infections in 1000 *I. ricinus *ticks collected in the city of Hannover

	Larvae (% pos.)	Nymphs (% pos.)	Males (% pos.)	Females (% pos.)	Total adults (% pos.)	% Positives
No.	60	507	229	204	433	
DFM +	0	11 (2.2)	38 (16.6)	25 (12.3)	63 (14.5)	7.4
qPCR +	1 (1.7)	92 (18.1)	75 (32.8)	75 (37.8)	150 (34.6)	24.3
qPCR ?*	1	4	0	3	3	

*: Duplicates tested three times in qPCR with each time one positive and one negative sample.

**Table 2:** Mono-, double and triple-infections of ticks with species of the *B. burgdorferi *sl-complex

	Larvae (%)	Nymphs (%)	Males (%)	Females (%)	Total adults (%)
No. of qPCR pos./?	2	96	75	78	153
Species not determined	1 (50.0)	19 (19.8)	9 (12.0)	4 (5.1)	13 (8.5)
Mono-infection	1 (50.0)	62 (64.6)	44 (58.7)	47 (60.3)	91 (59.5)
Double-infection	-	15 (15.6)	20 (26.7)	26 (33.3)	46 (30.1)
Triple-infection	-	-	2 (2.7)	1 (1.3)	3 (2.0)

**Table 3:** *Borrelia *species distribution in 251 positive/questionable ticks of the city of Hanover

Mono-infections	No. (%)	Double-infections	No. (%)	Triple-infections	No. (%)
*B. afzelii *(a)	52 (20.7)	a/b	6 (2.4)	a/b/s	1 (0.4)
*B. burgdorferi s.s*. (b)	11 (4.4)	a/g	10 (4.0)	g/s/v	2 (0.8)
*B. garinii *(g)	50 (20.0)	a/s	12 (4.8)		
*B. spielmanii *(s)	15 (6.0)	a/v	1 (0.4)		
*B. valaisiana *(v)	26 (10.4)	b/g	2 (0.8)		
Not determined	33 (13.2)	b/s	1 (0.4)		
		b/v	1 (0.4)		
		g/s	14 (5.6)		
		g/v	12 (4.8)		
		v/s	2 (0.8)		

### Analysis of *Borrelia *infections in ticks collected from hedgehogs

The 238 ticks collected from hedgehogs were represented by 92 nymphs and 146 adult females. All were morphologically identified as *I. hexagonus*. Genus-specific qPCR was successfully performed with 232 individuals of which 12 (5.7%) were positive for *B. burgdorferi *sl. These 12 positive ticks were composed of 2 nymphs and 10 adult females resulting in 2.17% positive nymphs and 6.85% positive adult females.

With species-specific conventional PCR in 4 ticks the *Borrelia *species could be determined. Two ticks were infected with *B. afzelii*, one with *B. spielmanii*, and one was double infected with *B. afzelii *and *B. garinii*.

## Discussion

Spirochetes of the *B. burgdorferi *sl-complex can be detected by dark-field and phase-contrast microscopy, Giemsa-stained smears, direct and indirect immunofluorescence, cultivation, and PCR methods. Detection of *Borrelia *infections in ticks is commonly done by dark-field microscopy (DFM) or PCR methods. However, DFM is less specific than PCR because all spirochete bacteria are diagnosed as positive, not exclusively *Borrelia *spp. Concerning sensitivity PCR is superior to DFM since successful DNA analysis does not require living organisms whereas DFM and also cultivation are dependent on living bacteria. But sensitivity is also varying among different PCR methods of which quantitative real time PCR (qPCR) is superior to conventional PCR. The established genus-specific *B. burgdorferi *sl-qPCR is based on probes enhancing specificity through a third oligonucleotide that has to match the target sequence. The designed TaqMan™ minor groove binder (MGB)-probe has an advantage over a conventional TaqMan™ probe regarding mismatch discrimination [22] and the use of a non-fluorescent quencher (NFQ) results in more precise fluorescence signal detection compared to dye quenchers. In conclusion, qPCR utilizing TaqMan™ MGB-probe is the most sensitive and specific detection system. The chosen target sequence, the 5S-23S intergenic spacer (IGS), represents a single copy region within the *B. burgdorferi *sl genome [20]. Thus, a reliable quantification of the *Borrelia *burden per tick or the like is given. Consistent with the sensitivity of a LightCycler hybridization probe based qPCR targeting the *osp*A gene of *B. burgdorferi *ss, *B. afzelii*, and *B. garinii *described by Rauter et al. [23], the detection limit of the established qPCR targeting all European genospecies was 1-10 copies. This corresponds to a detection limit of ≥50-500 *Borrelia *spirochetes per tick taken the volumes of eluted genomic DNA and template into account. To exclude that negative qPCR results follow from failed DNA isolations, a duplex qPCR was established targeting the *Ixodes *ITS2 sequence alongside the *B. burgdorferi *sl IGS sequence. The designed primer/probe combination detected the considered vector species *I. ricinus*, *I. hexagonus *and *I. canisuga*, which feed on humans and domestic mammals besides their wild-living hosts. However, *I. hexagonus *and *I. canisuga *are found less frequently parasitizing non wildlife species due to their nest adapted life cycle [24]. Noteworthy, duplex qPCR *Borrelia *detection was successful using the Thermo Fisher ABsolute™ Blue QPCR low Rox Mix but failed with the Stratagene Brilliant^® ^QPCR Master Mix.

From 1000 *I. ricinus *collected in recreation areas of the city of Hanover (Lower Saxony, Northern Germany) 24.3% were positive for *B. burgdorferi *sl considering all developmental tick stages. Similar infection rates (22%) were found in Southern Germany [17], whereas in Central Germany (Thuringia) only 11.1% were positive [14]. Interestingly, both authors observed with 9.0 and 8.6% almost identical *Borrelia *infections in nymphs. In contrast, in the present study the number of infected nymphs was with 18.1% nearly doubled. Also a higher percentage of adults were positive in the present study (34.6%) than in Central Germany (21.0%) and Southern Germany (29.7%). With respect to larvae, the number of positives is comparable to these of Southern Germany (1.7% and 1.5%; no data for Central Germany). Infections in larvae can either result from transovarial transmission [25,26], or, since this transmission is rather inefficient, from a blood meal that was interrupted and thus too short for nymph development.

In contrast to 24.3% positives in qPCR, DFM revealed only 7.4% positive ticks depicting the lower sensitivity of this method due to dependency on living spirochetes. This necessity of living bacteria and thus ticks for examination restricts also operational capacity, which is another disadvantage of the DFM technique. Furthermore, as mentioned above, DFM has a low specificity by reason that it is not possible to differentiate between *Borrelia *spp. and other spirochetes. This became apparent in 20 ticks that were spirochete positive in DFM but negative in genus-specific *B. burgdorferi *sl qPCR.

The most prevalent genospecies in the present study was *B. garinii *closely followed by *B. afzelii*. This is consistent with the findings of Hildebrandt et al. [14] and Fingerle et al. [17] whereas Rauter et al. [23] and Maetzel et al. [16] found *B. afzelii *more frequent than *B. garinii*. Interestingly, *B. burgdorferi *ss, the main causative genospecies of LB in the United States, was the third most common genospecies in Southern and Central Germany [14,17] but the least frequent in the presented study site in Northern Germany. These prevalence results could potentially be influenced by cross reactions with *B. bavariensis *sp. nov. since this species was not included in species-specific *rpo*B PCR specificity tests by Lee et al. [21]. For *B. lusitaniae *and *B. bissettii *as further species of the *B. burgdorferi *sl complex occurring in Central Europe the authors excluded cross reactions by amplification experiments. The present study revealed that the same is true for *B. spielmanii*. Regarding the *B. spielmanii osp*A specific primer pair, the present study negated cross reactions with *B. burgdorferi *ss, *B. afzelii*, *B. garinii *and *B. valaisiana *by PCR experiments. Also for *B. bissettii *and *B. lusitaniae osp*A primer sequence comparison gave strong indication that cross reactions can not occur whereas for *B. bavariensis *sp. nov. no sequence data where available. Consequently, amplification of this *Borrelia *species by *B. spielmanii osp*A primers could potentially occur.

In 33 out of the 251 positive/questionable ticks the *Borrelia *species could not be determined. One explanation is that the target sequence copy number was to low to produce visible bands in gel electrophoresis. Furthermore, *B. lusitaniae*, *B. bissettii *and *B. bavariensis *sp. nov. were not included in conventional species-specific PCR and thus could be considered as not determined species. However, *B. lusitaniae *is perpetuated by lizards and thus distributed mainly in Mediterranean countries [27]. In Germany, Fingerle et al. [17] found only 1 from 475 positive ticks collected at different study sites in Southern Germany to be infected with *B. lusitaniae*. The authors also identified *B. bissettii *in a human cerebrospinal fluid from Germany. Otherwise, this species is mainly reported in human patients from Slovenia [28] and Czech Republic [12,13]. In Czech Republic, 0.5% ticks were found to be infected with *B. bissettii *and 0.8% with *B. lusitaniae *[29]. The recently proposed new species *B. bavariensis *sp. nov. was so far described by Margos et al. [7] in human samples from Bavaria (Germany), Villach (Austria), and Slovenia as well as by Gern et al. [30] in xenodiagnostic ticks fed on 2 *Apodemus sylvaticus *mice from the Staatswald in Switzerland [31]. Thus, a small proportion of the unidentified tick infections may be due to *B. bissettii*, *B. lusitaniae *or and *B. bavariensis *sp. nov., but in most of them PCR failed most likely due to insufficient template amounts.

Infections with more than one genospecies were observed in nearly one third of the positive ticks. Because adult ticks had one more blood meal than nymphs, it is not surprising that the number of multiple infected adults (32.1%) is doubled compared to nymphs (15.6%). The combination *B. afzelii *and *B. spielmanii *as well as *B. garinii *and *B. valaisiana *made the most common double infections. For the latter ones birds are competent reservoirs [32,33]. Thus, bird-feeding ticks often are infected with both species. Kipp et al. [33] found that the blackbird and the song trush play an important role in the cycle of these two genospecies and at least blackbirds are very frequent in recreational or human inhabited areas. The combination *B. garinii/B. valaisiana *was also identified by Maetzel et al. [16] as the most common tick double infections. Interestingly, *B. spielmanii *was observed two times more in multiple infections (30 infected ticks, 12% of all infected ticks) than as single infection (15 infected ticks, 6% of all infected ticks).

Besides *I ricinus*, *I. hexagonus *ticks collected from hedgehogs were examined for *Borrelia *infection resulting in 5.7% positives. Identified *Borrelia *species were *B. afzelii*, *B. garinii *and *B. spielmanii*. Former studies revealed 2.6 and 2.7% positive *I. hexagonus *using DFM and direct immunofluorescence, respectively [2,34]. In a conventional PCR based study, 11.5% of *I. hexagonus *collected from hedgehogs were infected with *B. burgdorferi *sl [35]. Nest adaptation of *I. hexagonus *suggests that the ticks in the present study were infected by hedgehogs, for which reservoir competency for the *B. burgdorferi *sl-complex was shown [25,36].

## Conclusions

With the TaqMan™ MGB-probe based qPCR a sensitive, specific, and rapid tool to detect spirochetes of the *B. burgdorferi *sl-complex was established. Enhancement to a duplex qPCR also targeting the 16S region of tested common ixodid ticks provides a positive control of successful isolation of DNA from the ticks besides the diagnostic purpose, assuring proper results not only for epidemiological studies but also for statements of diagnostic laboratories processing transmitted ticks from private parties. The designed *B. spielmanii *specific conventional PCR filled the gap to identify the most common European pathogenic *Borrelia *species by DNA amplification since Lee et al. [21] described PCR protocols for *B. burgdorferi *ss, *B. afzelii, B. garinii *and *B. valaisiana *only.

We have found that all pathogenic species of the *B. burgdorferi *sl-complex were present in ticks collected in recreational areas in the city of Hanover and *B. afzelii*, *B. garinii *and *B. spielmanii *were identified in *I. hexagonus *ticks collected from hedgehogs confirming their role as reservoirs for pathogenic species of the *B. burgdorferi *sl-complex.

## Methods

### Spirochete cultures for subsequent DNA isolation

Spirochetes were cultured to obtain genomic DNA for establishing the genus- and species-specific *Borrelia *PCR and to determine sensitivity and specificity. Furthermore, the DNA of different species of the *Borrelia burgdorferi *sl-complex served as species-specific PCR positive controls. The following organisms were cultured: *B. burgdorferi *ss (strain B31 and LW2), *B. afzelii *(strain NE632 and ZQ1), *B. garinii *(strain 1B29 and A87SB), *B. valaisiana *(strain VS116), *B. spielmanii*, *Treponema sokranskii*, *Leptospira grippothyphosa*, and *L. interrogans*. These spirochetes were cultivated in sterile filtered modified Barbour-Stoenner-Kelly medium (BSKH medium, Sigma-Aldrich, Taufkirchen, Germany) at 37°C in 50 ml culture flasks (Sarstedt, Nürmbrecht, Germany). Cultures were monitored twice a week with the dark-field microscope, and refilled with 15 ml of fresh medium. After 14 days the cultures were centrifuged at 8000 × *g *for 10 min and the resulting pellets diluted with 50 μl of sterile filtered PBS. Genomic DNA was isolated using the QIAamp^®^ DNA Blood Mini Kit (Quiagen, Hilden), according to the manufacturer's instructions, and eluted in 100 μl of deionized H_2_O.

### Genus-specific quantitative real time PCR to detect *Borrelia burgdorferi *sl

For genus-specific detection of the *B. burgdorferi *sl-complex, a TaqMan™ minor groove binder (MGB)-probe and corresponding primers were designed using the Primer Express™ software (Applied Biosystems, Darmstadt, Germany). As target sequence the 5S-23S intergenic spacer (IGS) was chosen and the design based on species recognized in Europe, whereby diversity was considered in sequence selection. Selected sequences were (GenBank accession numbers and origin in brackets): *B. burgdorferi *ss (AF497981, Czech Republic; DQ393308, France; AY583237, Russia), *B. afzelii *(AF497984, Czech Republic; DQ111066, France; DQ020300, Russia), *B. garinii *(AF497993, Czech Republic; AY163784; Latvia; AY163784, Russia), *B. valaisiana *(AF497988, Czech Republic; AF497989, Czech Republic; U78150, The Netherlands), and *B. lusitaniae *(AB091801, Turkey; AB091799, Turkey; DQ111065, France).

Designed primer sequences and corresponding TaqMan™-MGB-probe were also checked for sequence matching with *B. spielmanii *(AF497994, Czech Republic; AM160603, Germany; AM183337, France) and *B. bissettii *(FJ431140, Czech Republic; FJ431142, Czech Republic; EF015627 USA) after sequence availability in the National Center for Biotechnology Information (NCBI) database. Primer and probe nucleotide sequences are listed in Table 4. The probe was purchased from Applied Biosystems (Darmstadt, Germany) and primers from Invitrogen (Karlsruhe, Germany).

**Table 4:** Primers and probes used for genus-specific quantitative real time PCR, species-specific conventional PCR and generation of plasmid standards

Primer/Probe	Sequence (5' to 3')	Amplicon size
IGS-MGB *Borrelia *for	TCC TAG GCA TTC ACC ATA GAC T	67 bp
IGS-MGB *Borrelia *rev	TGG CAA AAT AGA GAT GGA AGA T	
IGS-MGB *Borrelia *probe	6-FAM-ATT ACT TTG ACC ATA TTT-MGBNFQ	
		
*rpoB B. burgdorferi *s.s. for*	CTG TTG GTG AGC TTC TTA CT	308 bp
*rpoB B. burgdorferi *s.s. rev*	TCT ACC ATA ATG AGT ATA ATG C	
*rpoB B. afzelii *for*	AGA GTG CGT TCT GTT GGC	318 bp
*rpoB B. afzelii *rev*	TCT ACC ATA ATG AGT ATA ATG T	
*rpoB B. garinii *for*	GTG CGT TCT GTT GGG GAG	257 bp
*rpoB B. garinii *rev*	AGT CCC CCT GGT CCA AGG	
*rpoB B. valaisiana *for*	AGG AGA GTA CGT TCT GTT GGA	306 bp
*rpoB B. valaisiana *rev*	ATA ATG GAC GTC TCT TAC TTC A	
*ospA B. spielmanii *for	CAG TAG ATG TAC CTG GGG AAC TT	146 bp
*ospA B. spielmanii *rev	GCT TTT ACG CCT TCC AGT ACA	
		
ITS2-MGB *Ixodes *for	TGC GTC GTA GCC TTC	77 bp
ITS2-MGB *Ixodes *rev	AAC GGC ATT CCC CTA C	
ITS2-MGB *Ixodes *probe	6-VIC-TCT AAG ACC TTC GCG-MGBNFQ	

*: Primer sequences described by Lee et al. [21].

Performance of primers and probe in quantitative real time PCR (qPCR) was tested with isolated DNA from the above mentioned *Borrelia *cultures. The reaction was set up using the Brilliant^® ^QPCR Master Mix (Stratagene, Heidelberg, Germany): 8.49 μl deionized H_2_O, 12.50 μl Brilliant buffer (containing SureStart^® ^*Taq *Polymerase), 0.75 μl forward and reverse primer (100 μM each), respectively, 0.13 μl probe (50 μM), 0.38 μl diluted ROX as reference dye (1:500 dilution), and 2 μl template. Thermal cycling conditions were: 10 min at 94°C followed by 40 cycles of 20 sec at 94°C, 60 sec at 56°C and 45 sec at 72°C. Experiments and data analysis were performed using the Mx3005 Multiplex Quantitative PCR System (Stratagene, Heidelberg, Germany). Amplification products were analyzed via gel electrophoresis (2% agarose gels). The bands were cut out, ligated into pCR^® ^4-TOPO^® ^vector followed by transformation of *Escherichia coli *One Shot^® ^TOP 10 cells (TOPO TA Cloning^® ^Kit for Sequencing; Invitrogen, Karlsruhe, Germany). Plasmid DNA was obtained using the NucleoSpin^® ^Plasmid Kit (Macherey-Nagel, Dueren, Germany) following the manufacturer's recommendations and sequenced at the SEQLAB Sequence laboratories (Göttingen, Germany). Sequences were verified by using BLAST searching the non-redundant NCBI database http://www.ncbi.nlm.nih.gov/BLAST/.

The plasmid containing the verified sequence of *B. afzelii *strain NE632 served as plasmid standard in prospective qPCR runs to ensure effective PCR amplification and detection of the fluorescence signal as well as to compile standard curves for determination of the IGS copy number and thus for determination of the *Borrelia *burden in individual ticks. For these standards, 10-fold serial plasmid dilutions ranging from 10^0 ^to 10^6 ^copies per reaction were prepared.

While the primer/probe combination perfectly matched the IGS sequence of *B. burgdorferi *ss, *B. afzelii*, *B. garinii*, *B. bissettii*, and *B. valaisiana*, there was one mismatch in position 8 of the reverse primer sequence (C instead of T) with *B. spielmanii *and *B. lusitaniae*. To examine whether this mismatch has an impact on detection, qPCR runs were performed using a plasmid standard containing the targeted IGS-sequence of *B. spielmanii *and *B. lusitaniae *as template.

### Specificity and sensitivity of the genus-specific quantitative real time PCR

To test specificity, isolated DNA of cultured related spirochetes (*Treponema *sp. and *Leptospira *spp.) was checked for cross reactivity in qPCR runs. Besides this control, genomic DNA of 100 laboratory bred *I. ricinus *larvae was also used as negative control. These ticks were processed in the same manner as field-collected ticks described below.

Sensitivity testing was conducted by amplification of the plasmid standard serially diluted from 10^0 ^to 10^6 ^copies. Furthermore, DNA of different dilutions from cultured *B. afzelii *spirochetes (strain NE632) as well as spiked negative laboratory bred larvae, nymphs, and adults of *I. ricinus *were amplified to investigate the effect of tick material on the sensitivity of the qPCR.

### Duplex genus-specific quantitative real time PCR including a DNA isolation positive control

For verification of successful genomic DNA isolation, a duplex quantitative real time PCR was established targeting the ITS2-sequence of *Ixodes*-ticks besides the IGS-sequence of the *B. burgdorferi *sl-complex. The positive control primer/TaqMan™-MGB-probe combination was designed based on the ITS2-sequence of *I. ricinus *(GenBank accession number D88884) using the AlleleID^® ^7 software (version 7.01., Premier Biosoft International, Palo Alto, USA). The nucleotide sequences of the primers and corresponding TaqMan™-MGB-probe are listed in Table 4. The probe was purchased from Applied Biosystems (Darmstadt, Germany) and primers from Invitrogen (Karlsruhe, Germany). To ensure detection of the three most common mammalian *Ixodes *spp. feeding on mammals in Central Europe (*I. ricinus*, *I. hexagonus *and *I. canisuga*), the primer-probe combination was tested on genomic DNA of the following individuals: 280 *I. ricinus *(9 larvae, 134 nymphs, 71 adult males, and 66 adult females), 8 *I. hexagonus *(adult females), and 8 *I. canisuga *(1 larvae and 7 adult females).

To confirm qPCR signal detection, the genomic DNA of each eight individuals of the three *Ixodes *spp. was amplified with different primer combinations covering different lengths of the *Ixodes *ITS2-sequence. Used forward primers were: 5'-TTC TTT TGG CGT GGA TGT TGT TCG-3', 5'-TGC GTC GTA GCC TTC-3', and 5'-CTT CTT GCT CGA AGG AGA G-3'. Used reverse primers were: 5'-GCA TCG CTT TCG ATT CGA CAA AAA-3' and 5'-GGG GGT TGT CTC GCC TGA TGT G-3'. Nucleotide data obtained by sequencing the PCR products cloned into pCR^® ^4-TOPO^® ^have been submitted to the GenBank database under accession numbers GQ330512 - GQ330535. The sequence deposited under the acc. no. GQ330512 (*I. ricinus *amplified with the primer combination 5'-TTC TTT TGG CGT GGA TGT TGT TCG-3' and 5'-GGG GGT TGT CTC GCC TGA TGT G-3') represented the positive control plasmid standard in subsequent qPCR runs.

The set up for the genus-specific qPCR was adapted insofar as for the volume of *Ixodes *ITS2-specific primers and probe the amount of deionized H_2_O was reduced. Besides the used Brilliant^® ^QPCR Master Mix (Stratagene, Heidelberg, Germany), the ABsolute™ Blue QPCR low Rox Mix (Thermo Fisher Scientific, Hamburg, Germany) was tested for qPCR setup. The qPCR temperature profile was retained unchanged.

### Species-specific conventional PCR to analyse *B. burgdorferi *sl species distribution

Conventional species-specific PCRs can be used to differentiate species of the *B. burgdorferi *sl-complex. It was performed for each collected tick (described below) positive in genus-specific qPCR. The species *B. burgdorferi *ss, *B. afzelii, B. garinii *and *B. valaisiana *were detected with previously described specific primers targeting the RNA polymerase subunit B (*rpo*B) gene [21]. To detect *B. spielmanii*, outer surface protein A (*osp*A) gene specific primers were designed using the Lasergene PrimerSelect program (DNASTAR, version 5.06; GATC Biotech, Konstanz, Germany) as primer design software. The following *osp*A sequences from European *B. spielmanii *isolates were used for primer design: Accession nos. DQ133517 (France), AF102057 (The Netherlands), and EU545183 (Turkey). The nucleotide sequences of all species-specific primers supplied by Invitrogen (Karlsruhe, Germany) are listed in Table 4. Specificity of the designed *osp*A primers as well as the published *rpo*B primers was tested with DNA from *B. spielmanii*, *B. burgdorferi *ss, *B. afzelii, B. garinii*, and *B. valaisiana*, and other related spirochetes (*Treponema *spp. and *Leptospira *spp.) cultured as described above. For *B. bissettii *and *B. lusitaniae *specificity was checked via sequence alignment (Align Plus 5, vs. 5.04; Scientific & Educational software, Cary, USA) based on acc nos. DQ393323 and EF457558, respectively. Sequence information for *B. bavariensis *sp. nov. *osp*A were not available in the databases. Furthermore, specificity was tested on 100 laboratory bred *I. ricinus *larvae and additionally via Nucleotide BLAST http://blast.ncbi.nlm.nih.gov/Blast.cgi. The PCR product resulting from *B. spielmanii *DNA amplification was cloned and sequenced for verification.

PCR setup for the conventional species-specific PCR was as follows: 3 μl template was added to 14.2 μl deionized H_2_O, 2.5 μl 10× buffer, 2.5 μl 10× CoralLoad PCR buffer, 0.5 μl MgCl_2 _(50 mM), 0.5 μl deoxyribonucleotide triphosphates (10 mM each), 0.8 μl gene-specific forward and reverse primer (10 μM each), respectively, and 0.2 μl *Taq *Polymerase (5 U/μl; Qiagen, Hilden, Germany). PCR cycling (40 cycles) was performed using for *B. burgdorferi *ss, *B. afzelii, B. garinii *and *B. valaisiana *the annealing temperatures described by Lee et al. [21] and 57°C for *B. spielmanii*. The following temperature profile was used: Initial denaturing at 94°C for 4 min, denaturing at 94°C for 30 sec, annealing for 30 sec, extending primers at 72°C for 30 sec and final elongation for 4 min.

### Analysis of *B. burgdorferi *sl infections in ticks of the city of Hanover

In the city of Hanover, the capital of the federal state Lower Saxony, 1000 ticks were collected in recreation areas using the flagging method for subsequent investigation of infection with the *B. burgdorferi *sl-complex. Furthermore, the dark-field microscopy (DFM) technique should be compared to the established qPCR method.

To perform the DFM analysis, every selected tick was placed in an individual tube and mechanically homogenized with single-use polystyrene homogenizers (Roth, Karlsruhe, Germany) in 50 μl sterile filtered phosphate buffered saline (PBS; pH 7.2). From the homogenized solution, 10 μl were placed on a microscope slide and entirely examined for spirochetes using a DFM microscope (Zeiss, Jena, Germany). If one spirochete was found, the sample was determined as positive. After DFM analysis, the vials containing the ticks' homogenized solution were stored at -75°C until DNA isolation as described above.

When performing genus-specific qPCR, the micro titre plates contained duplicates of each tick sample as well as serially diluted plasmid standards to generate standard curves and a no-template control. Each tick positive in genus-specific qPCR was examined with species-specific conventional PCR to obtain information about the species distribution.

### Analysis of *Borrelia *infections in ticks collected from hedgehogs

Besides hanoverian ticks, 238 ticks collected from hedgehogs in 23 different sites of the federal states Schleswig-Holstein, North-Rhine Westphalia, Saxony, Baden-Württemberg, Bavaria, and Berlin were analysed for infection with *B. burgdorferi *sl by genus-specific quantitative real time PCR and positive ticks were analysed for species distribution via conventional PCR.

## Competing interests

The authors declare that they have no competing interests.

## Authors' contributions

VMM collected the ticks, carried out the molecular work and participated in analysis. EE carried out duplex quantitative real time PCR and participated in analysis. TS and CE conceived and designed the study and participated in analysis. CS drafted the manuscript, analysed and interpreted the data, and participated in the design of the study. All authors read and approved the final manuscript.
